# Interactions of zearalanone, α-zearalanol, β-zearalanol, zearalenone-14-sulfate, and zearalenone-14-glucoside with serum albumin

**DOI:** 10.1007/s12550-020-00404-w

**Published:** 2020-08-01

**Authors:** Zelma Faisal, Virág Vörös, Eszter Fliszár-Nyúl, Beáta Lemli, Sándor Kunsági-Máté, Miklós Poór

**Affiliations:** 1 Department of Pharmacology, Faculty of Pharmacy, University of Pécs, Szigeti út 12, Pécs, H-7624 Hungary; 2 János Szentágothai Research Centre, Ifjúság útja 20, Pécs, H-7624 Hungary; 3 Institute of Organic and Medicinal Chemistry, Medical School, University of Pécs, Szigeti út 12, Pécs, H-7624 Hungary

**Keywords:** Zearalanone, Zearalanols, Zearalenone-14-sulfate, Zearalenone-14-glucoside, Serum albumin, Species differences

## Abstract

The xenoestrogenic mycotoxin zearalenone is a *Fusarium*-derived food and feed contaminant. In mammals, the reduced (e.g., zearalanone, α-zearalanol, and β-zearalanol) and conjugated (e.g., zearalenone-14-sulfate) metabolites of zearalenone are formed. Furthermore, filamentous fungi and plants are also able to convert zearalenone to conjugated derivatives, including zearalenone-14-sulfate and zearalenone-14-glucoside, respectively. Serum albumin is the dominant plasma protein in the circulation; it interacts with certain mycotoxins, affecting their toxicokinetics. In a previous investigation, we demonstrated the remarkable species differences regarding the albumin binding of zearalenone and zearalenols. In the current study, the interactions of zearalanone, α-zearalanol, β-zearalanol, zearalenone-14-sulfate, and zearalenone-14-glucoside with human, bovine, porcine, and rat serum albumins were examined, employing fluorescence spectroscopy and affinity chromatography. Zearalanone, zearalanols, and zearalenone-14-sulfate form stable complexes with albumins tested (*K* = 9.3 × 10^3^ to 8.5 × 10^5^ L/mol), while the albumin binding of zearalenone-14-glucoside seems to be weak. Zearalenone-14-sulfate formed the most stable complexes with albumins examined. Considerable species differences were observed in the albumin binding of zearalenone metabolites, which may have a role in the interspecies differences regarding the toxicity of zearalenone.

## Introduction

Zearalenone (ZEN) is a xenoestrogenic mycotoxin produced by *Fusarium* species. It appears as a contaminant in crops, cereal-based products (e.g., flour, bakery goods, and beer), and in other commodities (Rogowska et al. 2019). Despite its nonsteroidal structure, ZEN can bind to estrogen receptors (Loi et al. 2017; Shier et al. 2001) causing reproductive disorders, as well as its potential genotoxic, hepatotoxic, teratogenic, and immunotoxic effects are also suggested (Rai et al. 2019; Rogowska et al. 2019). The involvement of ZEN in the development of breast and esophageal cancers has been emerged; however, ZEN is classified as a group 3 carcinogen by the IARC (Rai et al. 2019).

**Fig. 1:**
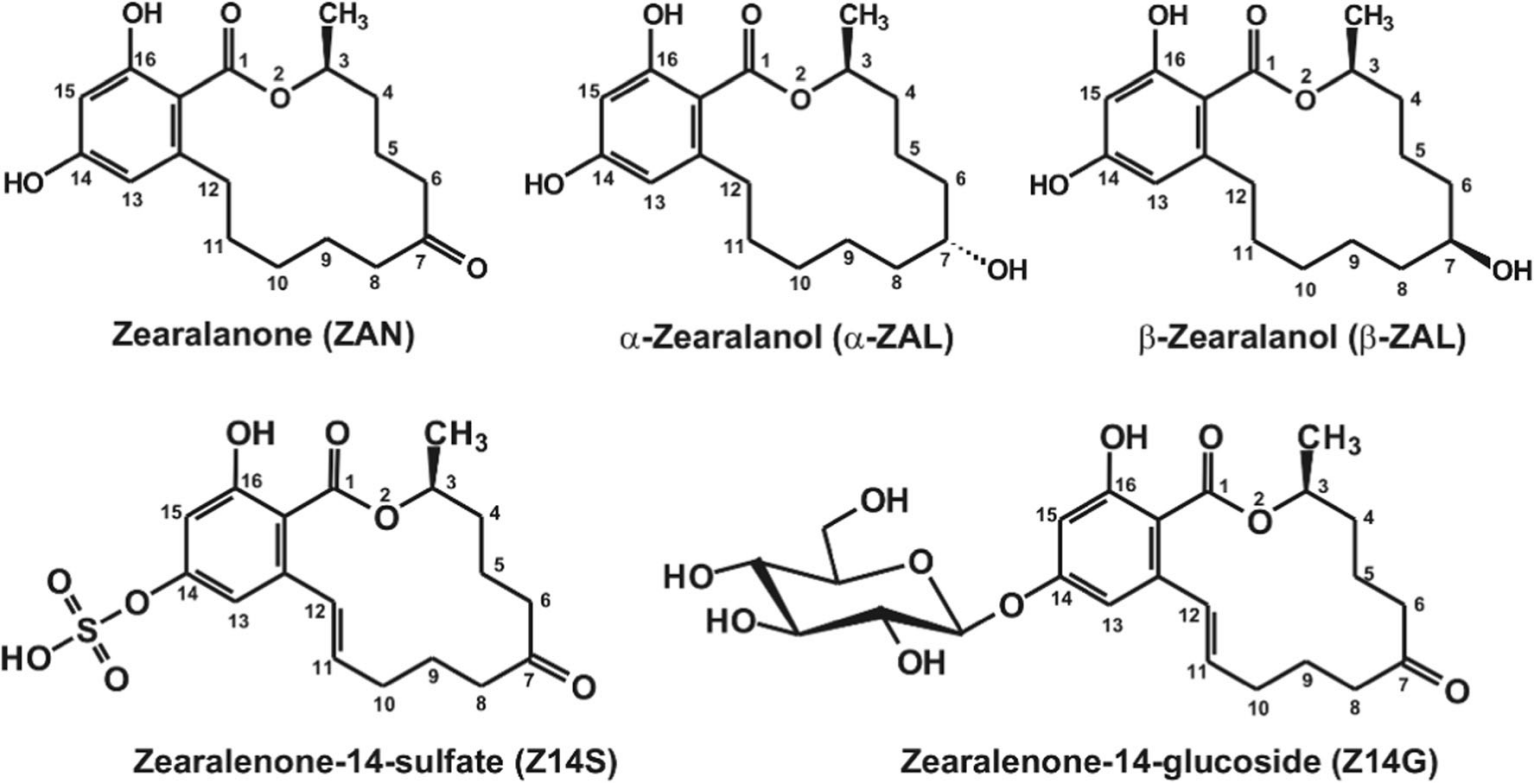
Chemical structures of zearalanone (ZAN), α-zearalanol (α-ZAL), β-zearalanol (β-ZAL), zearalenone-14-sulfate (Z14S), and zearalenone-14-glucoside (Z14G)

ZEN is extensively biotransformed in mammals (Rai et al. 2019). Its reduction by hydroxysteroid dehydrogenases leads to the formation of α- and β-zearalenols (α- and β-ZELs), zearalanone (ZAN, Fig. 1), and α- and β-zearalanols (α- and β-ZALs, Fig. 1) (EFSA 2017). Some of these metabolites (e.g., α-ZEL and α-ZAL) show considerably higher xenoestrogenic effects than ZEN (EFSA 2017; Fleck et al. 2017; Frizzell et al. 2011; Filannino et al. 2011). Furthermore, α-ZAL (also known as zeranol) is administered as a growth promoter to farm animals, leading to the appearance of the residual α-ZAL in food, mainly in beef (Mukherjee et al. 2014; EFSA 2017). Therefore, this application of α-ZAL is prohibited in the EU (while it is still available in some countries/regions, such as North America, Chile, Australia, New Zealand, South Africa, and Japan) (Mukherjee et al. 2014). In addition, the exposure to ZEN and α-ZAL may be responsible for the more frequent development of precocious puberty among young girls (Mukherjee et al. 2014).

As a result of the phase II metabolism of ZEN, the glucuronic acid and sulfate conjugates of ZEN, ZELs, and ZALs are produced, such as zearalenone-14-glucuronide and zearalenone-14-sulfate (Z14S; also known as zearalenone-4-sulfate; Fig. 1) (Mirocha et al. 1981; Olsen et al. 1986; Dänicke and Winkler 2015; EFSA 2017; Warth et al. 2019). In mammals, glucuronide conjugates take part in enterohepatic circulation, during which they are cleaved in the intestines, and the reabsorption of the deconjugated products considerably increases their elimination half-life (Dänicke et al. 2005; EFSA 2017).

Plants and filamentous fungi can also modify the structures of parent mycotoxins (Berthiller et al. 2013). Mycotoxin derivatives formed during these reactions, and via other biological or chemical degradation processes, are classified as modified mycotoxins (Rychlik et al. 2014; Freire and Sant’Ana 2018). Among modified mycotoxins, the plant-produced conjugates are called masked mycotoxins (Rychlik et al. 2014). Plants and/or filamentous fungi can convert ZEN to Z14S (molar ratios of ZEN to Z14S are between 12:1 and 2:1); therefore, the latter compound appears as a food contaminant (Freire and Sant’Ana 2018; Plasencia and Mirocha 1991; Berthiller et al. 2006). The amount of Z14S in cereal-based products showed high variations (1–417 μg/kg), depending on the product tested and the year of harvest (Vendl et al. 2010; De Boevre et al. 2012). Zearalenone-14-glucoside (Z14G; also known as zearalenone-4-glucoside; Fig. 1) is an abundant plant-produced conjugate of ZEN, which has been found in ZEN-contaminated grain-based products (Berthiller et al. 2013; Vendl et al. 2010). Typically, in vitro models demonstrate the lower toxicity of ZEN conjugates compared with the parent mycotoxin. Previous in vitro studies described that the glucoside conjugation of ZEN prevents the binding of these derivatives to the human estrogen receptors (Poppenberger et al. 2006), and the significantly lower (100-fold) estrogenicity of Z14S vs. ZEN has been also reported (Drzymala et al. 2015). However, the gut microbiota can hydrolyze these metabolites, leading to the formation of ZEN (Berthiller et al. 2013; EFSA 2017). After the oral administration of Z14S and Z14G to pigs, their complete intestinal hydrolysis to ZEN was observed (Binder et al. 2017). Therefore, the EFSA evaluation suggests that the risks regarding the exposure to Z14S and Z14G should be considered similar to ZEN (EFSA 2017). The European Food Safety Authority (EFSA) CONTAM Panel established human tolerable daily intake (TDI) of 0.25 μg/kg body weight per day, denoted as ZEN equivalents for ZEN and its modified forms jointly (EFSA 2014).

Serum albumin is the major protein in the circulation; it binds to several endogenous compounds and xenobiotics. The complex formation can affect the tissue distribution and the elimination half-life of ligand molecules (Fanali et al. 2012; Yamasaki et al. 2013). ZEN and ZELs form stable complexes with serum albumin, showing considerable species differences (Poór et al. 2017; Ma et al. 2018; Faisal et al. 2018). For example, the affinity of ZEN and ZELs towards rat albumin is approximately tenfold higher compared with albumins from other species (Faisal et al. 2018). The differences in albumin binding may be partly responsible for the high variations in the toxicokinetics of ZEN and its derivatives, and may help to understand the vulnerability of some species vs. these mycotoxins. Cirlini et al. reported the absorption and partial deglycosylation of Z14G in an in vitro model with CaCo-2 cells, the absorption of Z14G was considerably lower compared with ZEN (Cirlini et al. 2016). After the oral administration of Z14G to rats, its low plasma concentrations were detected (Sun et al. 2019). Furthermore, approximately 61% of the orally administered Z14G was absorbed in pigs, which was followed by the significant presystemic hydrolysis of the masked mycotoxin (Catteuw et al. 2019). These data indicate that a lower fraction of Z14G can reach the systemic circulation. A previous study suggests the interaction of Z14G with human serum albumin (HSA), during which albumin can slowly hydrolyze the masked mycotoxin to ZEN (Dellafiora et al. 2017).

In this study, the interactions of ZAN, α-ZAL, β-ZAL, Z14S, and Z14G with human (HSA), bovine (BSA), porcine (PSA), and rat (RSA) serum albumins were investigated by fluorescence spectroscopy. Furthermore, to confirm the results of spectroscopic studies, the interactions of ZEN metabolites with HSA were also examined with high-performance affinity chromatography (HPAC).

## Materials and methods

### Reagents

Zearalenone (ZEN; MW = 318.36 g/mol; purity: 99.7%, HPLC), zearalanone (ZAN; MW = 320.38 g/mol; purity: 100%, TLC ), α-zearalanol (α-ZAL; MW = 322.40 g/mol; purity: 98%, HPLC), β-zearalanol (β-ZAL; MW = 322.40 g/mol; purity: 98%, HPLC), HSA (MW = 66.4 kDa), BSA (MW = 66.4 kDa), PSA (MW = 67.5 kDa), RSA (MW = 64.6 kDa), and warfarin (MW = 308.3 g/mol) were obtained from Sigma-Aldrich (Saint Louis, MO, USA). Zearalenone-14-sulfate ammonium salt (Z14S; MW = 415.46 g/mol; purity: 98.5%, HPLC, NMR, LC-MS) was purchased from ASCA GmbH (Berlin, Germany). Zearalenone-14-*O*-β-d-glucoside (Z14G; MW = 480.50 g/mol; purity: 99.4%, HPLC, NMR) was obtained from Honeywell (Charlotte, NC, USA). Stock solutions of mycotoxins (5000 μmol/L; ZEN: 1.592 g/L; ZAN: 1.602 g/L; ZALs: 1.612 g/L; Z14S ammonium salt: 2.078 g/L; and Z14G: 2.403 g/L) were prepared in ethanol (96 v/v%, spectroscopic grade; VWR, Debrecen, Hungary) and stored at – 20 °C.

### Spectroscopy

Fluorescence spectroscopic measurements were carried out employing a Hitachi F-4500 fluorescence spectrophotometer (Hitachi, Tokyo, Japan) to investigate the effect of increasing mycotoxin concentrations on the fluorescence signal of albumins as well as on the emission spectrum of warfarin-HSA complex. Our studies were executed in phosphate-buffered saline (PBS, pH 7.4; 8.00 g/L NaCl, 0.20 g/L KCl, 1.81 g/L Na_2_HPO_4_ × 2H_2_O, 0.24 g/L KH_2_PO_4_) at room temperature, in the presence of air.

For spectral correction of fluorescence emission intensities, absorption spectra of mycotoxins were also recorded applying a Jasco-V730 spectrophotometer (Jasco, Tokyo, Japan). The inner-filter effect of mycotoxins was corrected as described previously (Hu and Liu 2015; Faisal et al. 2018):

1 $$ {I}_{\mathrm{cor}}={I}_{\mathrm{obs}}\times {e}^{\left({A}_{\mathrm{ex}}+{A}_{\mathrm{em}}\right)/2} $$

where *I*_cor_ and *I*_obs_ indicate the corrected and observed fluorescence emission intensities, respectively. *A*_ex_ and *A*_em_ denote the absorbance of mycotoxins at the excitation and emission wavelengths used, respectively.

To investigate the stability of mycotoxin-albumin complexes, mycotoxin-induced quenching effects on the intrinsic fluorescence of albumins were tested. The emission signal of albumins (2 μmol/L; *λ*_ex_ = 295 nm; *λ*_ex_ = 340 nm) were measured in the presence of increasing concentrations of mycotoxins (0.0, 1.0, 2.0, 3.0, 4.0, 5.0, 6.0, 7.0, 8.0, 9.0, and 10.0 μmol/L). Stern-Volmer quenching constants (*K*_SV_; unit: L/mol) were determined employing the Stern-Volmer equation (Ma et al. 2018; Faisal et al. 2018):

2 $$ \frac{I_0}{I}=1+{K}_{\mathrm{SV}}\times \left[Q\right] $$

where *Q* is the concentration of the mycotoxin (unit: mol/L). *I*_0_ and *I* are the fluorescence emission signal of albumin in the absence and presence of mycotoxins, respectively. Z14S and Z14G exert fluorescence; their excitation and emission spectra, under different environmental conditions, have been reported previously (Faisal et al. 2019, 2020). Under the applied circumstances, the emission signals of Z14S and Z14G did not interfere with the evaluation of fluorescence studies (e.g., the emission maxima of albumins and warfarin-HSA complex). Furthermore, ZAN and ZALs did not exert fluorescence at the concentrations applied.

Binding constants (*K*; unit: L/mol) of mycotoxin-albumin complexes were determined by nonlinear fitting employing Hyperquad2006 software, as described in details in our previous studies (Sueck et al. 2018; Faisal et al. 2018).

To test the effects of ZEN metabolites on warfarin-HSA interaction, our previously reported method was applied (Faisal et al. 2018; Fliszár-Nyúl et al. 2019). In this experiment, the fluorescence emission signal of warfarin (1 μmol/L; *λ*_ex_ = 317 nm, *λ*_em_ = 379 nm) was examined in the presence of HSA (3.5 μmol/L) without and with mycotoxins (0, 1, 2, 3, 4, 5, 6, 8, 10, and 15 μmol/L) in PBS (pH 7.4). Under these conditions, approximately 70% of warfarin is albumin-bound. Since albumin-bound warfarin shows much higher fluorescence than the free fluorophore (Faisal et al. 2018), the changes in its fluorescence can indicate the increased or decreased albumin binding of warfarin.

### High-performance affinity chromatography

HPAC was performed with a HSA-coated column (Faisal et al. 2018). The HPLC system (Jasco, Tokyo, Japan) used for the analysis included an autosampler (AS-4050), a binary pump (PU-4180), and a diode-array detector (MD-4017). A 5-μL volume of samples (ZAN: 200 μmol/L; ZEN, α-ZAL, β-ZAL, and Z14S: 100 μmol/L; Z14G: 50 μmol/L) was driven through a pre-column filter (Waters, Milford, MA, USA) linked to an immobilized HSA-coated HPAC column (Chiralpak^®^ HSA, 50 × 3.0 mm, 5 μm; Daicel, Tokyo, Japan). The isocratic elution was performed with 0.5-mL/min flow rate at room temperature. The mobile phase contained isopropanol (HPLC grade; VWR, Debrecen, Hungary) and 0.01 mol/L pH 7.0 ammonium acetate buffer (15:85 v/v%). Mycotoxins were detected at 235 nm, and chromatograms were evaluated with ChromNAV software.

## Results and discussion

### Fluorescence quenching studies

**Fig. 2:**
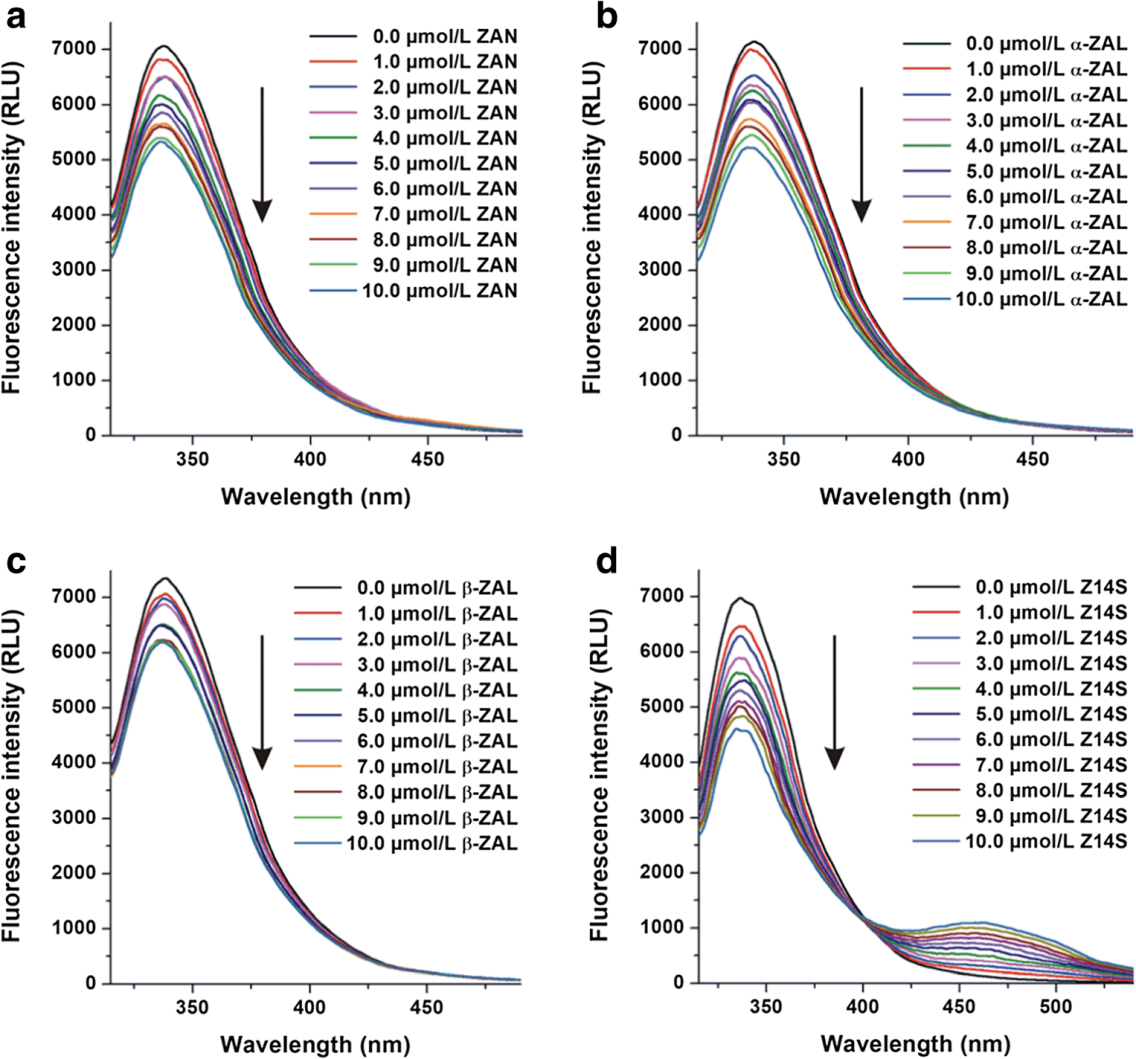
Fluorescence quenching effects of ZEN metabolites on HSA. Emission spectra of HSA (2 μmol/L) in the presence of increasing concentrations of ZAN, α-ZAL, β-ZAL, and Z14S in PBS (pH 7.4; *λ*_ex_ = 295 nm). Z14G did not affect the emission spectra of albumins (ZAN zearalanone, α-ZAL α-zearalanol, β-ZAL β-zearalanol, Z14S zearalenone-14-sulfate, Z14G zearalenone-14-glucoside)

In this experiment, the fluorescence quenching effects of ZAN, ZALs, Z14S, and Z14G (0-10 μmol/L each) on albumins (2 μmol/L) were investigated in PBS, using 295 nm excitation wavelength. Under these circumstances, albumins showed their emission wavelength maxima around 340 nm. Inner-filter effects of ZEN metabolites were eliminated employing Eq. 1. Z14G did not affect the emission signals of albumins (data not shown); therefore, it is reasonable to hypothesize that Z14G does not interact or forms only low-affinity complexes with albumins. However, other mycotoxins tested induced concentration-dependent decrease in the fluorescence of albumins at 340 nm (Fig. 2), suggesting the formation of albumin-ligand complexes (Tan et al. 2019; Ma et al. 2018; Faisal et al. 2018; Fliszár-Nyúl et al. 2019). The strongest quenching effect was shown by Z14S, and an increasing second peak appeared in these spectra at approximately 460 nm (Fig. 2d), which is the fluorescence signal of Z14S. Despite the fact that ZEN and ZELs exert intrinsic fluorescence (Faisal et al. 2018), ZAN and ZALs showed negligible fluorescence under the applied conditions. Therefore, no secondary peaks appeared in Fig. 2a–c.

**Fig. 3:**
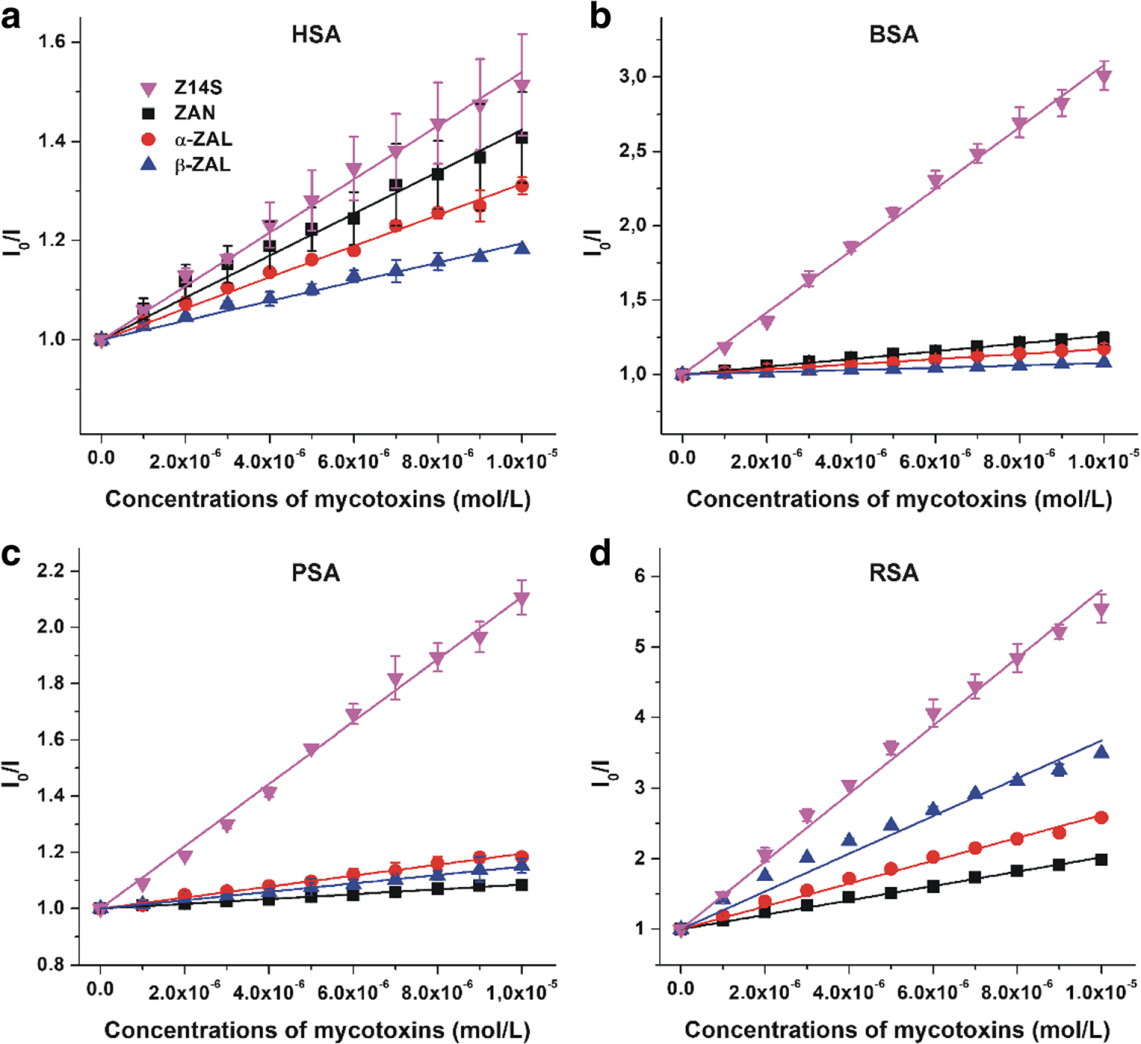
Stern-Volmer plots (*R*^2^ = 0.969–0.997) of mycotoxin-albumin interactions: HSA (**a**), BSA (**b**), PSA (**c**), and RSA (**d**) (*λ*_ex_ = 295 nm, *λ*_em_ = 340 nm; ZAN zearalanone, α-ZAL α-zearalanol, β-ZAL β-zearalanol, Z14S zearalenone-14-sulfate, HSA human serum albumin, BSA bovine serum albumin, PSA porcine serum albumin, RSA rat serum albumin)

Based on the mycotoxin-induced quenching effects, Stern-Volmer quenching constants (*K*_SV_, unit: L/mol; Table 1) and binding constants (*K*, unit: L/mol; Table 2) of albumin-ligand complexes were determined employing the graphical application of the Stern-Volmer equation (Eq. 2) and the Hyperquad2006 software, respectively. Stern-Volmer plots are demonstrated in Fig. 3, displaying good linearity for interactions tested (*R*^2^ = 0.97–0.99). Hyperquad evaluation showed the best fitting with the 1:1 stoichiometry of complex formation; furthermore, good correlation of *K*_SV_ and *K* values was observed (see in Tables 1 and 2). ZEN metabolites tested (except Z14G) formed stable complexes with albumins, showing *K* values in a wide range (10^4^ to 10^6^ L/mol). Similarly to ZEN and ZELs (Faisal et al. 2018), mycotoxins formed the most stable complexes with RSA (Table 2). ZAN and ZALs bound with the lowest affinity to PSA or BSA, while Z14S formed the least stable complex with HSA among albumins tested. Furthermore, Z14S bound to each albumin with higher affinity than ZAN and ZALs, showing considerably stronger interactions with BSA and PSA compared with the reduced metabolites examined in this study. The binding constants of ZAN and ZALs showed minor differences regarding one individual albumin (Table 2). Typically, ZAN and ZALs formed less stable complexes with albumins than the parent compound ZEN; in contrast, Z14S-albumin displayed higher stability vs. ZEN-albumin complexes, except HSA (Faisal et al. 2018). Remarkable species-dependent differences were noticed regarding the albumin binding of ZAN, ZALs, and Z14S. For example, the binding affinity of ZAN-RSA vs. ZAN-PSA (14-fold), α-ZAL-RSA vs. α-ZAL-BSA (9-fold), β-ZAL-RSA vs. β-ZAL-BSA (30-fold), and Z14S-RSA and Z14S-HSA (16-fold) showed major differences (Table 2). Similarly, high species-dependent differences in albumin binding have been also reported regarding ZEN, ZELs, and ochratoxin A (Faisal et al. 2018; Hagelberg et al. 1989; Poór et al. 2014).

**Table 1:** Decimal logarithmic values of the Stern-Volmer quenching constants (*K*_SV_; unit: L/mol) of mycotoxin-albumin complexes

Mycotoxin*	log*K*_SV_ ± SEM			
	HSA	BSA	PSA	RSA
ZEN	5.09 ± 0.01^a^	4.81 ± 0.01^a^	4.56 ± 0.02^a^	5.50 ± 0.01^a^
ZAN	4.52 ± 0.04	4.41 ± 0.04	3.97 ± 0.06	5.00 ± 0.03
α-ZAL	4.50 ± 0.02	4.20 ± 0.05	4.30 ± 0.05	5.21 ± 0.00
β-ZAL	4.34 ± 0.04	3.88 ± 0.06	4.13 ± 0.09	5.43 ± 0.01
Z14S	4.64 ± 0.03	5.32 ± 0.02	5.04 ± 0.02	5.70 ± 0.02
Z14G	-	-	-	-

*ZEN zearalenone, *ZAN* zearalanone, *α-ZAL* α-zearalanol, *β-ZAL* β-zearalanol, *Z14S* zearalenone-14-sulfate, *Z14G* zearalenone-14-glucoside, *HSA* human serum albumin, *BSA* bovine serum albumin, *PSA* porcine serum albumin, *RSA* rat serum albumin

^a^Based on Faisal et al. (2018)

**Table 2:** Decimal logarithmic values of binding constants (*K*; unit: L/mol) of mycotoxin-albumin complexes

Mycotoxin*	log*K* ± SEM			
	HSA	BSA	PSA	RSA
ZEN	5.09 ± 0.01^a^	4.78 ± 0.01^a^	4.57 ± 0.01^a^	5.42 ± 0.00^a^
ZAN	4.58 ± 0.00	4.51 ± 0.00	3.97 ± 0.01	5.12 ± 0.00
α-ZAL	4.55 ± 0.00	4.34 ± 0.00	4.38 ± 0.00	5.31 ± 0.01
β-ZAL	4.37 ± 0.01	4.12 ± 0.01	4.15 ± 0.01	5.61 ± 0.01
Z14S	4.71 ± 0.03	5.43 ± 0.02	5.12 ± 0.02	5.93 ± 0.02
Z14G	-	-	-	-

*ZEN zearalenone, *ZAN* zearalanone, *α-ZAL* α-zearalanol, *β-ZAL* β-zearalanol, *Z14S* zearalenone-14-sulfate, *Z14G* zearalenone-14-glucoside, *HSA* human serum albumin, *BSA* bovine serum albumin, *PSA* porcine serum albumin, *RSA* rat serum albumin

^a^Based on Faisal et al. (2018)

The toxicokinetics of ZEN show large variances in different species, which may be partly resulted from the species-dependent alternations in albumin binding of ZEN and its metabolites (Fruhauf et al. 2019). Some of the recent in vivo studies support this hypothesis (Fruhauf et al. 2019; Catteuw et al. 2019; Mukherjee et al. 2014). In pigs, the lower affinity of the mycotoxin towards PSA (Z14G < β-ZEL < α-ZEL < ZEN) was accompanied with its earlier disappearance from the circulation (Catteuw et al. 2019; Faisal et al. 2018), which propose the potential impact of albumin binding on the toxicokinetics of these mycotoxins. Furthermore, the significantly longer plasma elimination half-lives of ZEN and α-ZAL have been reported in rats vs. in humans (Mukherjee et al. 2014), which is in agreement with the considerably higher affinity of ZEN (Faisal et al. 2018) and α-ZAL (Table 2) towards RSA compared to HSA.

### Elution of ZEN and its metabolites from HSA-HPAC column

**Fig. 4:**
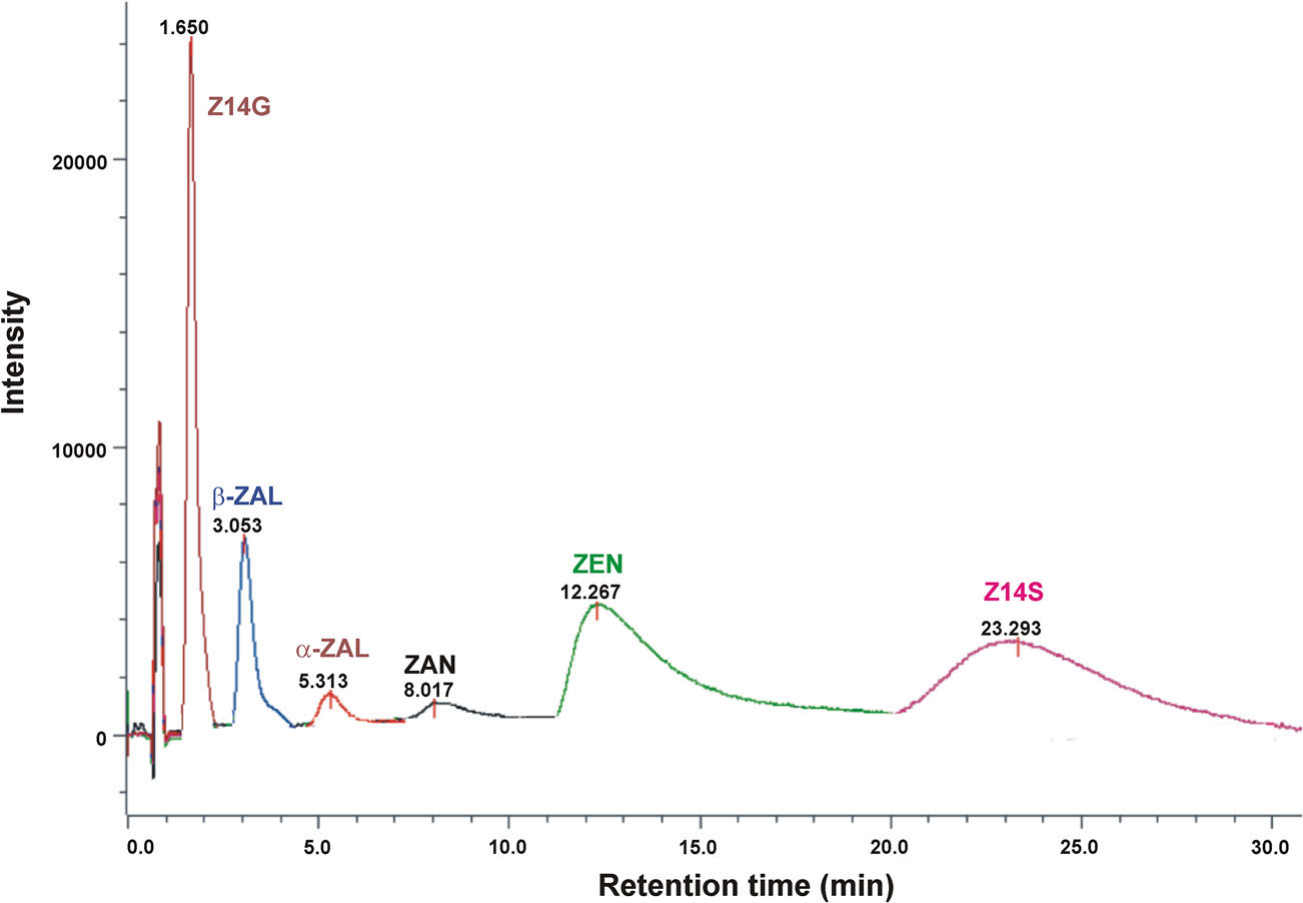
HPAC chromatograms of ZEN, ZAN, α-ZAL, β-ZAL, Z14S, and Z14G eluted from the HSA-coated column (see details in “High-performance affinity chromatography” section; ZAN zearalanone, α-ZAL α-zearalanol, β-ZAL β-zearalanol, Z14S zearalenone-14-sulfate, Z14G zearalenone-14-glucoside)

To confirm the results of quenching studies, the interactions of ZEN, ZAN, ZALs, Z14S, and Z14G with HSA were also examined employing HPAC. The stronger interaction of the ligand molecule with HSA leads to its longer elution from the HSA-HPAC column. The mycotoxins tested were eluted with the following retention times (*t*_R_) from the affinity column (Fig. 4): Z14G (1.7 min), β-ZAL (3.1 min), α-ZAL (5.3 min), ZAN (8.0 min), ZEN (12.3 min), and Z14S (23.3 min). The *t*_R_ of Z14G was very short but it was not eluted with the solvent front, indicating the weak interaction of Z14G with HSA. The formation of low-affinity Z14G-HSA complexes is in agreement with the previously reported very slow hydrolysis of Z14G by the protein (Dellafiora et al. 2017). The *t*_R_ of ZEN metabolites (Fig. 4) suggests the same orders in complex stability than quenching studies (Table 2): Z14S > ZAN > α-ZAL > β-ZAL > Z14G. Furthermore, the longer *t*_R_ of ZEN vs. ZAN, ZALs, and Z14G are also in agreement with the current results (Table 2) and previous observations (Faisal et al. 2018). However, the *t*_R_ of Z14S was even longer compared with ZEN, despite its binding affinity is lower (log*K*_Z14S-HSA_ = 4.7; log*K*_ZEN-HSA_ = 5.1) based on quenching studies (Faisal et al. 2018). This discrepancy may be explained by the different experimental conditions in quenching and HPAC studies, which can influence the stability of albumin-ligand complexes (Kaspchak et al. 2018). In quenching studies, PBS (pH 7.4) was applied to mimic extracellular physiological condition. However, in the HPAC studies, we created appropriate conditions for the affinity column (based on the manufacturer’s guide); therefore, the buffer was different, the ionic strength and the pH were lower than in quenching studies, and the eluent contained isopropanol (see details in the “Materials and methods” section).

### Effects of ZEN metabolites on warfarin-HSA interaction

**Fig. 5:**
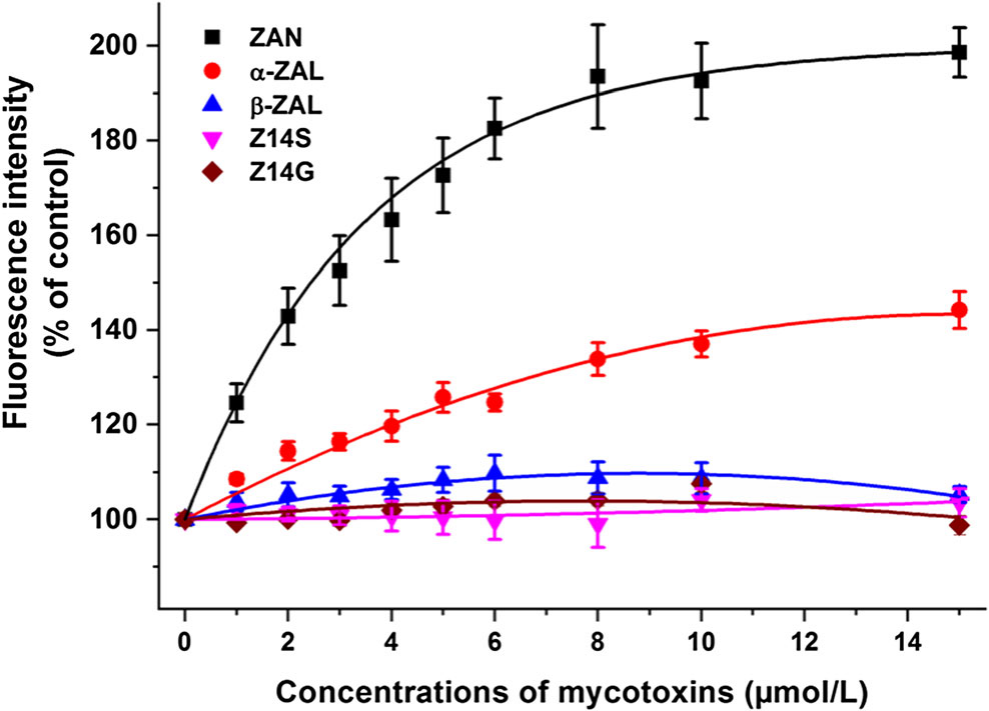
Allosteric effects of ZEN analogs on the binding of warfarin to HSA. Fluorescence emission signal of warfarin (1 μmol/L) with HSA (3.5 μmol/L) in the presence of increasing mycotoxin concentrations in PBS (pH 7.4; *λ*_ex_ = 317 nm, *λ*_em_ = 379 nm; ZAN zearalanone, α-ZAL α-zearalanol, β-ZAL β-zearalanol, Z14S zearalenone-14-sulfate, Z14G zearalenone-14-glucoside; the inner-filter effects of mycotoxins were corrected based on Eq. 1)

In previous studies, the allosteric interactions of ZEN and ZELs with the Sudlow’s site I ligand warfarin have been reported (Poór et al. 2017; Faisal et al. 2018): Since the binding sites of ZEN and ZELs are relatively close to the site I region, these mycotoxins can increase (ZEN and α-ZEL) or decrease (β-ZEL) the binding affinity of warfarin towards HSA. Therefore, the effects of ZAN, ZALs, Z14S, and Z14G on warfarin-HSA interaction were also examined. Because albumin-bound warfarin exerts considerably higher fluorescence signal at 379 nm than free warfarin, the increase or decrease in the fluorescence at 379 nm indicate its elevated or reduced albumin binding, respectively (Faisal et al. 2018; Fliszár-Nyúl et al. 2019). Importantly, the inner-filter effects of mycotoxins were also corrected in these experiments (see Eq. 1). As Fig. 5 demonstrates, ZAN and α-ZAL considerably increased the emission signal of warfarin, similar to ZEN and α-ZEL in our previous study (Faisal et al. 2018). These observations suggest that ZAN and α-ZAL can increase the binding affinity of warfarin towards HSA. However, β-ZAL, Z14S, and Z14G did not affect the fluorescence at 379 nm. Because Z14G forms low-affinity complexes with HSA, it is not surprising that it did not modify the albumin binding of warfarin. In our previous study, β-ZEL showed different effect compared with ZEN and α-ZEL, likely due to its different binding position or binding site (Faisal et al. 2018). Therefore, the observation that β-ZAL and Z14S have no effect on warfarin-HSA interaction (despite their binding affinities are similar to ZAN and α-ZAL) suggests their different binding positions/sites compared with ZEN, ZAN, α-ZEL, and α-ZAL.

In conclusion, fluorescence spectroscopic and HPAC experiments suggest the weak interaction of Z14G with HSA; however, ZAN, ZALs, and Z14S form stable complexes with serum albumins investigated (*K* = 10^4^ to 10^6^ L/mol). In addition, significant species-dependent differences were observed in the affinity of ZAN, ZALs, and Z14S towards albumins from various species (human, bovine, porcine, and rat). These results suggest that albumin binding may be partly responsible for the species-dependent alterations in the toxicokinetics and toxic effects of ZEN and its metabolites previously described in mammals. For example, the formation of highly stable complexes of ZEN and α-ZAL with RSA can explain the long elimination half-lives of these mycotoxins in rat (Mukherjee et al. 2014). Furthermore, the binding constants determined in the current (Table 1) and previous (Faisal et al. 2018) studies show good correlation with the recently reported in vivo experiments performed in pigs (Catteuw et al. 2019): the higher binding constant causes the longer lifetime of the mycotoxin in the circulation. These observations underline the potential toxicokinetic importance of the albumin-ligand interactions of ZEN and its metabolites. Based on our data, it is reasonable to hypothesize that the albumin-bound fraction of ZEN derivatives is significant in the circulation; therefore, the appropriate sample preparation is highly important during the analyses of blood samples. In addition, the formation of stable mycotoxin-albumin complexes likely makes possible the application of albumin as an affinity protein for the extraction of ZEN and its metabolites, as it has been reported during the extraction of ochratoxin A with BSA from wine (Leal et al. 2019). Thus, these interactions may also have some analytical importance.

## Data Availability

We have full control of all primary data and we agree to allow the journal to review our data if requested.

## References

[CR1] Berthiller F, Crews C, Dall’Asta C, Saeger SD, Haesaert G, Karlovsky P, Oswald IP, Seefelder W, Speijers G, Stroka J (2013). Masked mycotoxins: a review. Mol Nutr Food Res.

[CR2] Berthiller F, Werner U, Sulyok M, Krska R, Hauser MT, Schuhmacher R (2006). Liquid chromatography coupled to tandem mass spectrometry (LC-MS/MS) determination of phase II metabolites of the mycotoxin zearalenone in the model plant *Arabidopsis thaliana*. Food Addit Contam.

[CR3] Binder SB, Schwartz-Zimmermann HE, Varga E, Bichl G, Michlmayr H, Adam G, Berthiller F (2017). Metabolism of zearalenone and its major modified forms in pigs. Toxins.

[CR4] Catteuw A, Broekaert N, De Baere S, Lauwers M, Gasthuys E, Huybrechts B, Callebaut A, Ivanova L, Uhlig S, De Boevre M, De Saeger S, Gehring R, Devreese M, Croubels S (2019). Insights into in vivo absolute oral bioavailability, biotransformation, and toxicokinetics of zearalenone, α-zearalenol, β-zearalenol, zearalenone-14-glucoside, and zearalenone-14-sulfate in pigs. J Agric Food Chem.

[CR5] Cirlini M, Barilli A, Galaverna G, Michlmayr H, Adam G, Berthiller F, Dall’Asta C (2016). Study on the uptake and deglycosylation of the masked forms of zearalenone in human intestinal Caco-2 cells. Food Chem Toxicol.

[CR6] Dänicke S, Swiech E, Buraczewska L, Ueberscha K (2005). Kinetics and metabolism of zearalenone in young female pigs. J Anim Physiol Anim Nutr.

[CR7] Dänicke S, Winkler J (2015). Invited review: diagnosis of zearalenone (ZEN) exposure of farm animals and transfer of its residues into edible tissues (carry over). Food Chem Toxicol.

[CR8] De Boevre M, Di Mavungu JD, Landschoot S, Audenaert K, Eeckhout M, Maene P, Haesaert G, De Saeger S (2012). Natural occurrence of mycotoxins and their masked forms in food and feed products. World Mycotoxin J.

[CR9] Dellafiora L, Galaverna G, Righi F, Cozzini P, Dall’Asta C (2017). Assessing the hydrolytic fate of the masked mycotoxin zearalenone-14-glucoside-a warning light for the need to look at the “maskedome”. Food Chem Toxicol.

[CR10] Drzymala SS, Binder J, Brodehl A, Penkert M, Rosowski M, Garbe L-A, Koch M (2015). Estrogenicity of novel phase I and phase II metabolites of zearalenone and cis-zearalenone. Toxicon.

[CR11] EFSA – European Food Safety Authority, Panel on contaminants in the food chain (2014) EFSA Panel on contaminants in the food chain, scientific opinion on the risks for human and animal health related to the presence of modified forms of certain mycotoxins in food and feed. EFSA J. 12:3916. 10.2903/j.efsa.2014.3916

[CR12] EFSA – European Food Safety Authority, Panel on contaminants in the food chain (2017) Risks for animal health related to the presence of zearalenone and its modified forms in feed. EFSA J 15:4851. 10.2903/j.efsa.2017.4851 10.2903/j.efsa.2017.4851 PMC7009830 32625539

[CR13] Faisal Z, Fliszár-Nyúl E, Dellafiora L, Galaverna G, Dall’Asta C, Lemli B, Kunsági-Máté S, Szente L, Poór M (2019). Cyclodextrins can entrap zearalenone-14-glucoside: interaction of the masked mycotoxin with cyclodextrins and cyclodextrin bead polymer. Biomolecules.

[CR14] Faisal Z, Fliszár-Nyúl E, Dellafiora L, Galaverna G, Dall’Asta C, Lemli B, Kunsági-Máté S, Szente L, Poór M (2020). Interaction of zearalenone-14-sulfate with cyclodextrins and the removal of the modified mycotoxin from aqueous solution by beta-cyclodextrin bead polymer. J Mol Liq.

[CR15] Faisal Z, Lemli B, Szerencses D, Kunsagi-Mate S, Balint M, Hetenyi C, Kuzma M, Mayer M, Poór M (2018). Interactions of zearalenone and its reduced metabolites α-zearalenol and β-zearalenol with serum albumins: species differences, binding sites, and thermodynamics. Mycotoxin Res.

[CR16] Fanali G, di Masi A, Trezza V, Marino M, Fasano M, Ascenzi P (2012). Human serum albumin: from bench to bedside. Mol Asp Med.

[CR17] Filannino A, Stout TA, Gadella BM, Sostaric E, Pizzi F, Colenbrander B, Dell’Aquila ME, Minervini F (2011). Dose-response effects of estrogenic mycotoxins (zearalenone, alpha- and beta-zearalenol) on motility, hyperactivation and the acrosome reaction of stallion sperm. Reprod Biol Endocrinol.

[CR18] Fleck SC, Churchwell MI, Doerge DR (2017). Metabolism and pharmacokinetics of zearalenone following oral and intravenous administration in juvenile female pigs. Food Chem Toxicol.

[CR19] Fliszár-Nyúl E, Lemli B, Kunsági-Máté S, Dellafiora L, Dall’Asta C, Cruciani G, Pethő G, Poór M (2019). Interaction of mycotoxin alternariol with serum albumin. Int J Mol Sci.

[CR20] Freire L, Sant’Ana AS (2018). Modified mycotoxins: an updated review on their formation, detection, occurrence, and toxic effects. Food Chem Toxicol.

[CR21] Frizzell C, Ndossi D, Verhaegen S, Dahl E, Eriksen G, Sørlie M, Ropstad E, Muller M, Elliott CT, Connolly L (2011). Endocrine disrupting effects of zearalenone, alpha- and beta-zearalenol at the level of nuclear receptor binding and steroidogenesis. Toxicol Lett.

[CR22] Fruhauf S, Novak B, Nagl V, Hackl M, Hartinger D, Rainer V, Labudová S, Adam G, Aleschko M, Moll W-D, Thamhesl M, Grenier B (2019). Biotransformation of the mycotoxin zearalenone to its metabolites hydrolyzed zearalenone (HZEN) and decarboxylated hydrolyzed zearalenone (DHZEN) diminishes its estrogenicity in vitro and in vivo. Toxins.

[CR23] Hagelberg S, Hult K, Fuchs R (1989). Toxicokinetics of ochratoxin A in several species and its plasma-binding properties. J Appl Toxicol.

[CR24] Hu T, Liu Y (2015). Probing the interaction of cefodizime with human serum albumin using multi-spectroscopic and molecular docking techniques. J Pharm Biomed Anal.

[CR25] Kaspchak E, Mafra LI, Mafra MR (2018). Effect of heating and ionic strength on the interaction of bovine serum albumin and the antinutrients tannic and phytic acids, and its influence on in vitro protein digestibility. Food Chem.

[CR26] Leal T, Abrunhosa L, Domingues L, Venâncio A, Oliveira C (2019). BSA-based sample clean-up columns for ochratoxin A determination in wine: method development and validation. Food Chem.

[CR27] Loi M, Fanelli F, Liuzzi VC, Logrieco AF, Mulè G (2017). Mycotoxin biotransformation by native and commercial enzymes: present and future perspectives. Toxins.

[CR28] Ma L, Maragos CM, Zhang Y (2018). Interaction of zearalenone with bovine serum albumin as determined by fluorescence quenching. Mycotoxin Res.

[CR29] Mirocha CJ, Pathre SV, Robison TS (1981). Comparative metabolism of zearalenone and transmission into bovine milk. Food Cosmet Toxicol.

[CR30] Mukherjee D, Royce SG, Alexander JA, Buckley B, Isukapalli SS, Bandera EV, Zarbl H, Georgopoulos PG (2014). Physiologically-based toxicokinetic modeling of zearalenone and its metabolites: application to the Jersey girl study. PLoS One.

[CR31] Olsen M, Mirocha CJ, Abbas HK, Johansson B (1986). Metabolism of high concentrations of dietary zearalenone by young male turkey poults. Poult Sci.

[CR32] Plasencia J, Mirocha C (1991). Isolation and characterization of zearalenone sulfate produced by *Fusarium* spp. Appl Environ Microbiol.

[CR33] Poór M, Kunsági-Máté S, Bálint M, Hetényi C, Gerner Z, Lemli B (2017). Interaction of mycotoxin zearalenone with human serum albumin. J Photochem Photobiol B.

[CR34] Poór M, Li Y, Matisz G, Kiss L, Kunsági-Máté S, Kőszegi T (2014). Quantitation of species differences in albumin-ligand interactions for bovine, human and rat serum albumins using fluorescence spectroscopy: a test case with some Sudlow’s site I ligands. J Lumin.

[CR35] Poppenberger B, Berthiller F, Bachmann H, Lucyshyn D, Peterbauer C, Mitterbauer R, Schuhmacher R, Krska R, Glössl J, Adam G (2006). Heterologous expression of *Arabidopsis* UDP-glucosyltransferases in *Saccharomyces cerevisiae* for production of zearalenone-4-O-glucoside. Appl Environ Microbiol.

[CR36] Rai A, Das M, Tripathi A (2019) Occurrence and toxicity of a fusarium mycotoxin, zearalenone. Crit Rev Food Sci Nutr:1–20. 10.1080/10408398.2019.1655388 10.1080/10408398.2019.1655388 31446772

[CR37] Rogowska A, Pomastowski P, Sagandykova G, Buszewski B (2019). Zearalenone and its metabolites: effect on human health, metabolism and neutralisation methods. Toxicon.

[CR38] Rychlik M, Humpf H-U, Marko D, Dänicke S, Mally A, Berthiller F, Klaffke H, Lorenz N (2014). Proposal of a comprehensive definition of modified and other forms of mycotoxins including “masked” mycotoxins. Mycotoxin Res.

[CR39] Shier WT, Shier AC, Xie W, Miroch CJ (2001). Structure-activity relationships for human estrogenic activity in zearalenone mycotoxins. Toxicon.

[CR40] Sueck F, Poór M, Faisal Z, Gertzen CGW, Cramer B, Lemli B, Kunsági-Máté S, Gohlke H, Humpf HU (2018). Interaction of ochratoxin A and its thermal degradation product 2’R-ochratoxin A with human serum albumin. Toxins.

[CR41] Sun F, Tan H, Li Y, De Boevre M, De Saeger S, Zhou J, Li Y, Rao Z, Yang S, Zhang H (2019). Metabolic profile, bioavailability and toxicokinetics of zearalenone-14-glucoside in rats after oral and intravenous administration by liquid chromatography high-resolution mass spectrometry and tandem mass spectrometry. Int J Mol Sci.

[CR42] Tan H, Chen L, Ma L, Liu S, Zhou H, Zhang Y, Guo T, Liu W, Dai H, Yu Y (2019). Fluorescence spectroscopic investigation of competitive interactions between quercetin and aflatoxin B_1_ for binding to human serum albumin. Toxins.

[CR43] Vendl O, Crews C, MacDonald S, Krska R, Berthiller F (2010). Occurrence of free and conjugated *Fusarium* mycotoxins in cereal based food. Food Addit Contam Part A.

[CR44] Warth B, Preindl K, Manser P, Wick P, Marko D, Buerki-Thurnherr T (2019). Transfer and metabolism of the xenoestrogen zearalenone in human perfused placenta. Environ Health Perspect.

[CR45] Yamasaki K, Chuang VT, Maruyama T, Otagiri M (2013). Albumin-drug interaction and its clinical implication. Biochim Biophys Acta.

